# Genetic evidence strengthens the connection between gut microbiota and gingivitis: a two-sample Mendelian randomization study

**DOI:** 10.3389/fcimb.2024.1380209

**Published:** 2024-05-15

**Authors:** Zhou Hang, Chen Rouyi, Li Sen

**Affiliations:** ^1^School & Hospital of Stomatology, Wenzhou Medical University, Wenzhou, China; ^2^The 1 School of Medicine, School of Information and Engineering, The 1 Affiliated Hospital of Wenzhou Medical University, Wenzhou, China; ^3^School of Basic Medical Sciences, Wenzhou Medical University, Wenzhou, China

**Keywords:** gut microbiota, gingivitis, Mendelian randomization, oral-gut axis, causal effect

## Abstract

**Introduction:**

The oral cavity and gut tract, being interconnected and rich in microbiota, may have a shared influence on gingivitis. However, the specific role of distinct gut microbiota taxa in gingivitis remains unexplored. Utilizing Mendelian Randomization (MR) as an ideal method for causal inference avoiding reverse causality and potential confounding factors, we conducted a comprehensive two-sample MR study to uncover the potential genetic causal impact of gut microbiota on gingivitis.

**Methods:**

Instrumental variables were chosen from single nucleotide polymorphisms (SNPs) strongly associated with 418 gut microbiota taxa, involving 14,306 individuals. Gingivitis, with 4,120 cases and 195,395 controls, served as the outcome. Causal effects were assessed using random-effect inverse variance-weighted, weighted median, and MR-Egger methods. For replication and meta-analysis, gingivitis data from IEU OpenGWAS were employed. Sensitivity analyses included Cochran’s Q tests, funnel plots, leave-one-out analyses, and MR-Egger intercept tests. This study aimed to assess the genetic correlation between the genetically predicted gut microbiota and gingivitis using linkage disequilibrium score regression (LDSC).

**Results:**

Three gut microbiota taxa (class Actinobacteria id.419, family Defluviitaleaceae id.1924, genus Defluviitaleaceae UCG011 id.11287) are predicted to causally contribute to an increased risk of gingivitis (P< 0.05). Additionally, four gut microbiota taxa (class Actinobacteria id.419, genus Escherichia Shigella id.3504, genus Ruminococcaceae UCG002 id.11360) potentially exhibit inhibitory causal effects on the risk of gingivitis (P< 0.05). No significant evidence of heterogeneity or pleiotropy is detected. Our findings indicate a suggestive genetic correlation between class Actinobacteria id.419, class Bacteroidia id.912, family Defluviitaleaceae id.1924, genus Escherichia Shigella id.3504 and gingivitis.

**Conclusion:**

Our study establishes the genetic causal effect of 418 gut microbiota taxa on gingivitis, offering insights for clinical interventions targeting gingivitis. Subsequent research endeavors are essential to corroborate the findings of our present study.

## 1 Introduction

Gingivitis is an inflammatory condition of the gingival tissue, most commonly caused by bacterial infection. Among all the periodontal diseases, gingivitis is considered to be the commonest. Gingivitis, as a significant public health issue, threatens thousands of people worldwide, imposing a considerable economic and health burden on society (Peres et al., 2019). The gut microbiota constitutes the largest microbial habitat in the human body, playing a pivotal role in metabolic and immunological functions. Consequently, any alterations in the gut microbiota can potentially lead to significant systemic repercussions (Boulange et al., 2016).

Recent studies have revealed that gut inflammation involves multiple pathways, with the gut microbiome playing a significant role in health (He et al., 2021). In the aspect of oral health, researcher proposed a concept of the ‘oral-gut axis’, emphasizing the interconnectedness between oral and gut microbiota (Chen B.Y. et al., 2023; Ferrillo et al., 2023). This interaction may also play a role in the crosstalk of systemic inflammation complications mediated by gingivitis (Van Dyke et al., 2020). Specifically, pathogenic organisms linked to gingivitis may impact the composition of the gut microbiota through the continuous swallowing of saliva, thereby influencing systemic diseases (Lam et al., 2023). On the other side, alterations in gut microbiota induced by systemic diseases are often concomitant with changes in oral microbiota and localized gingivitis lesions, affecting the host immune response (Byrd and Gulati, 2021).

Recent years have seen an exploratory study uncovering a connection between gut microbiota and gingivitis. Irrespective of periodontal conditions, a significant number of oral taxa associated with periodontal destruction and inflammation have been identified in the gut microbiomes of individuals (Lourenvarsigmao et al., 2018). Furthermore, gum therapy has been shown to effectively alleviate inflammatory symptoms in patients with gingivitis and associated systemic diseases, including notable improvement in those with liver cirrhosis (Bajaj et al., 2018). Some non-surgical periodontal therapy (NSPT) methods, such as incorporating oral probiotics, have been proposed as adjuncts to subgingival instrumentation. The administration of probiotics has shown beneficial effects, albeit limited, on clinical and microbiological outcomes in the management of Gingivitis patients (Ozener et al., 2023). Despite the limited clinical evidence supporting them, these initial studies underscore the importance of gut microbiota in gingivitis.

In the medical and therapeutic context, it’s essential to determine if the link between gut microbiota and gingivitis is merely correlational or driven by pathogenic mechanisms. Despite extensive research on epidemiology and pathophysiology, the causal association remains unclear due to reverse causality and other confounding effects. For example, experimental research has highlighted the role of Bacteroides in exacerbating gingivitis (Caselli et al., 2020). However, this stands in contrast to observational studies that propose the anti-inflammatory effects of Bacteroides metabolites, presenting a complex narrative (Luo et al., 2023). To navigate these complexities, MR offers a unique approach. MR employs the correlation between disease and genotype to simulate the influence of exposure factors on the disease, introducing genetic variations related to these factors as instrumental variables (Birney, 2022). Due to its temporal validity and capability to minimize confounding factors, Mendelian randomization is regarded as a supplementary approach to randomized controlled trials (Larsson et al., 2023).

In this study, we performed a two-sample MR analysis using publicly available genome-wide association study (GWAS) databases. Our goal was to investigate the potential causal relationship between gut microbiota and gingivitis, offering genetic evidence for the significance of gut microbiota in gingival health.

## 2 Materials and methods

### 2.1 Study design

**Figure 1:**
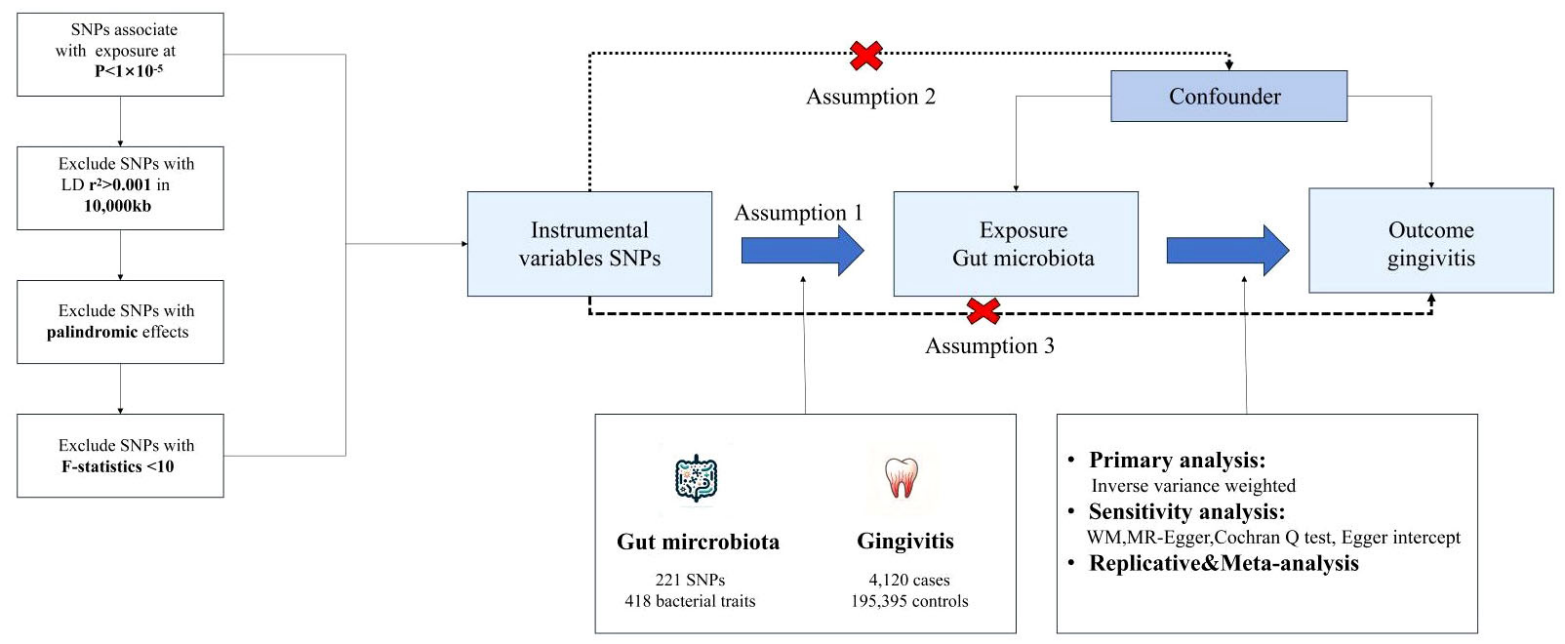
The core design and key assumptions of the present MR study. IVW inverse-variance weighted, the main analysis to investigate the association between exposure and outcome, LD linkage disequilibrium, it is used to calculate the correlations between SNPs; MR Mendelian randomization, SNP single nucleotide polymorphism, as genetic instrumental variables for the exposure and outcome.

We used a MR design with two samples to systematically evaluate the causal relationship between 418 gut microbiota and the risk of gingivitis. A robust MR design follows three basic assumptions: (1) genetic instruments are closely related to exposure; (2) Genetic tools are not associated with confounding factors; (3) Genetic tools only affect results through exposure of interest. The second and third hypotheses, collectively known as the independence of horizontal pleiotropy, are evaluated using various statistical methods. The genetic data for gingivitis was obtained from the FinnGen consortium for primary and IEU OpenGWAS for replication analysis, followed by meta-analysis (**Figure 1**).

### 2.2 Data source

The GWAS statistics for gingivitis in primary analysis were obtained from the FinnGen consortium, comprising 4,120 gingivitis cases and 195,395 control subjects, all of European descent and including both males and females, utilizing the human genome version HG19/GRCh37.

To validate our results by conducting replication analysis and meta-analysis, we used the gingivitis data from IEU OpenGWAS (acute gingivitis: 149 cases and 195,395 controls; chronic gingivitis: 850 cases and 195,395 controls), which is publicly available at the website: https://gwas.mrcieu.ac.uk/.

### 2.3 Instrument selection

The criteria for the 418 taxa included: (i) P< 1×10–^5^, since limited SNPs could be obtained under the genome-wide significance (P< 5×10–^8^) and such relaxed threshold have also been applied in many studies (Sanna et al., 2019); (ii) Independent SNPs (r ^2^< 0.001, distance > 10,000 kb) were preserved after calculating the linkage disequilibrium of related SNPs. Palindromic SNPs, characterized by alleles composed of a base and its complementary base, were also excluded from the analysis due to the potential confusion they might introduce regarding targeted alleles.

### 2.4 Statistical analyses

Firstly, R² was introduced to represent the proportion of phenotypic variance explained by SNPs (Equation 1) (Papadimitriou et al., 2020). Subsequently, F-statistics were cumulatively calculated to assess the strength of instrumental variables (IVs) (Equation 2). A threshold of F-statistic > 10 was considered indicative of robust statistical power, suggesting that the impact of weak instrument bias on the estimates of causal associations was unlikely (Burgess et al., 2011).


(1) $$R^{2}=2\times EAF\times \left(1-EAF\right)\times Beta^{2}$$

(2) $$F-statistic=\frac{n-k-1}{k}\times \frac{R^{2}}{1-R^{2}}$$

(Note: n, k, and EAF indicate the sample size, the number of IVs used, and effect allele frequency, respectively).

The main study utilized the inverse-variance weighted (IVW) approach, assuming the validity of all IVs and combining their effects to generate a weighted total effect (Brion et al., 2013). The primary analysis employed the random-effects inverse variance-weighted (IVW) method, as it is considered the most robust approach. This method can yield a moderate estimate even in the presence of heterogeneity. In cases of substantial heterogeneity (P<0.05), the random-effects model was implemented. Conversely, in the absence of significant heterogeneity (P>0.05), the fixed-effects model was employed. When a minimum of 50% of the weighted variance introduced by horizontal pleiotropy was valid, the weighted median (WM) estimates were considered capable of offering robust effect estimates (Bowden et al., 2016). To strengthen the robustness of causal conclusions, we utilized the MR pleiotropy residual sum and outlier (MR-PRESSO) test to identify and correct outliers potentially influenced by horizontal pleiotropy. This process entailed the removal of aberrant SNPs (Verbanck et al., 2018). The MR-Egger technique included an intercept term in the regression model to evaluate directional pleiotropy. A statistically significant non-zero intercept indicated the presence of pleiotropy, suggesting a deviation from the fundamental MR assumption (Bowden et al., 2015).

In addition, we performed various sensitivity analyses, including Cochran’s Q tests, funnel plots, leave-one-out analyses, and MR-Egger intercept tests. Cochran’s Q tests were specifically employed to detect heterogeneity, the MR-Egger intercept term was used to assess pleiotropy, and leave-one-out analyses were conducted to determine if any individual SNP significantly influenced the causal estimate.

A significance threshold of P<0.05 was utilized. Odds ratios (OR) with 95% confidence intervals (CI) were employed to illustrate the association between gut microbiota and gingivitis. The analyses were conducted using the “MendelianRandomization (version 0.7.0),” “MRPRESSO (version 1.0),” and “TwoSampleMR (version 0.5.7)” packages in R software (version 4.3.1).

### 2.5 Replication and meta−analysis

To validate the robustness of candidate metabolites, we replicated IVW analysis using another independent gingivitis GWAS data from IEU OpenGWAS the mentioned above, and then conducted a meta-analysis to determine the final candidates (**Figure 1**).

### 2.6 Genetic correlation validation

It’s been noted in the literature that Mendelian Randomization can lead to false-positive results due to genetic correlations among different traits (O’Connor and Price, 2018). In our approach, while SNPs directly linked to gingivitis were excluded during the selection of instruments, there remains the possibility that a group of SNPs, each individually not showing a significant association with gingivitis, could collectively contribute to its genetic predisposition. To explore whether the causative links we uncovered might be influenced by overlapping genetic factors, we conducted a LDSC analysis to examine the genetic correlations between the gut microbiota identified and gingivitis.

## 3 Results

### 3.1 Selection of instrumental variables

By applying a whole-genome significance threshold (P<1×10–^5^), conducting screening, performing LD testing, coordinating, and validating F-statistics, we identified multiple SNPs as IVs for each of the 418 bacterial taxa. All retained SNPs had F-statistics exceeding 10, indicating a robust correlation between the IVs and their corresponding bacterial taxa. Therefore, our study did not reveal evidence of weak instrumental bias (**Supplementary Materials Tables S1**, **S2**).

### 3.2 Causal effects of gut microbiota on gingivitis

**Figure 2:**
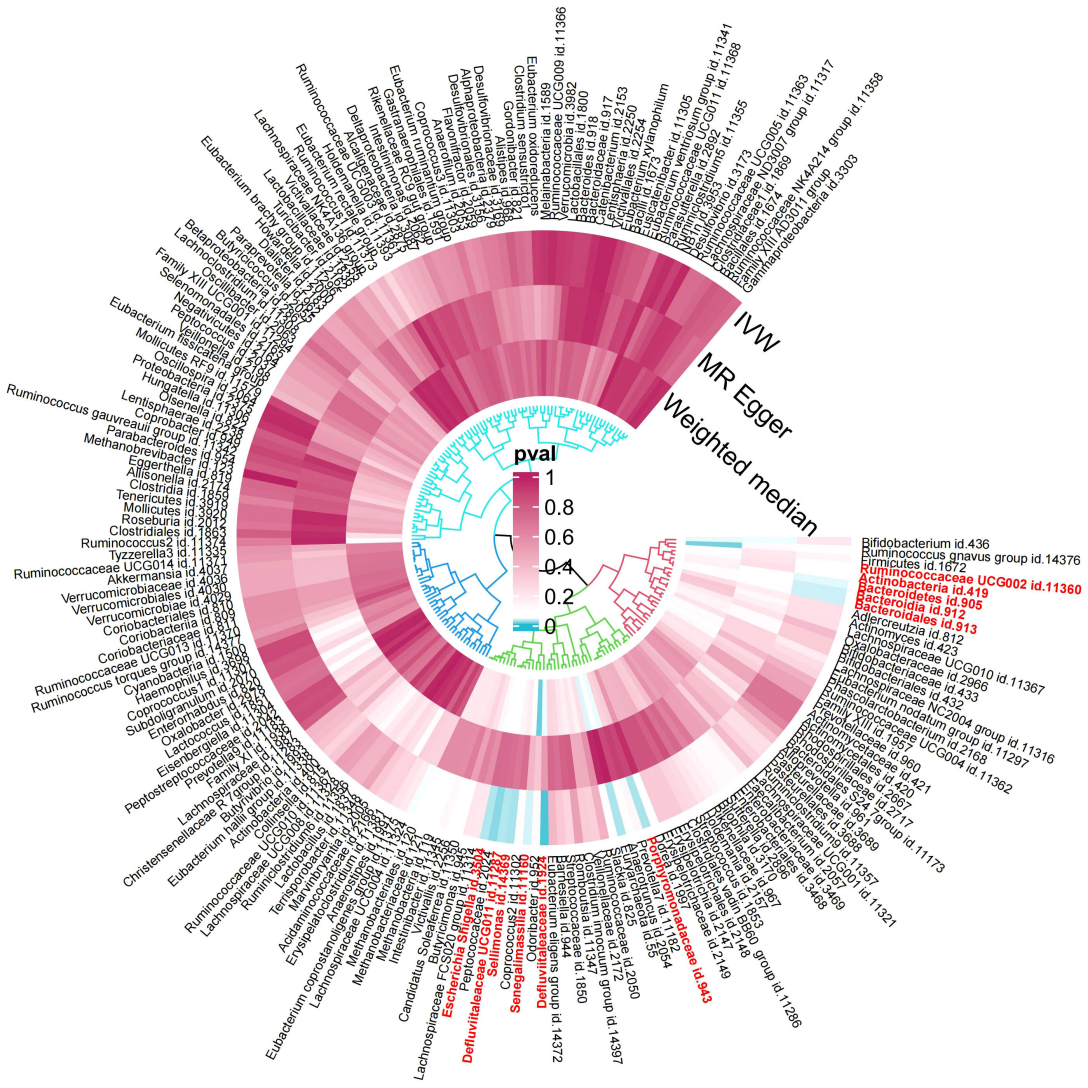
Preliminary MR analyses for the associations between gut microbiota and the risk of gingivitis. The circle from the outer to the inner represented the IVW, MR-Egger and WM estimates, respectively. The shades of color were reflections of the magnitude of the p-value as the label inside the circle (MR, Mendelian randomization; IVW, inverse variance-weighted; WM, weighted median).

In the exploration phase, we employed the IVW method for an initial investigation. Significant heterogeneity was not detected based on Cochran’s Q tests. Preliminary results for the analyses of associations between genetically proxied gut bacterial taxa and risks of gingivitis are as shown in **Figure 2**.

**Figure 3:**
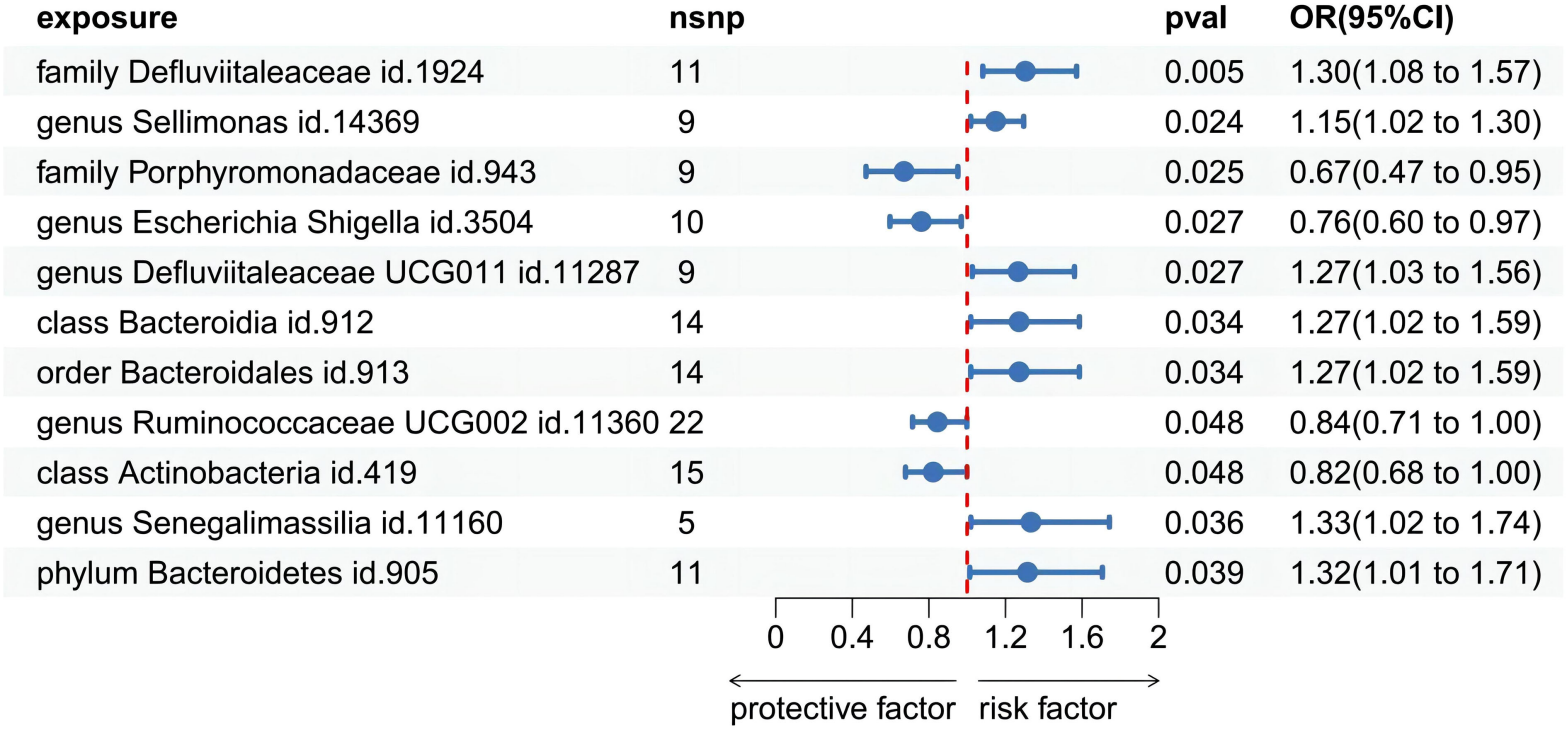
Forest plot of Mendelian randomization estimates between Gut microbiota and gingivitis. The figure showed the IVW estimates of significantly gingivitis -associated gut microbiota taxa. The blue dots represent the IVW estimates, and the blue bars represent the 95% confidence intervals of IVW estimates. The OR>1 indicates increased risk while<1 indicates decreased risk.

Among the 418 bacterial taxa, we find 11 gut microbiota taxa causally associated with gingivitis (**Figure 3**). As a result, we found that the family Defluviitaleaceae id.1924 (OR: 1.30, 95% CI 1.08–1.57, P=0.005), genus Sellimonas id.14369(OR: 1.14, 95% CI 1.01–1.29, P=0.02), genus Defluviitaleaceae UCG011 id.11287 (OR: 1.26, 95% CI 1.02–1.56, P=0.02), class Bacteroidia id.912 (OR: 1.27, 95% CI 1.02–1.58, P=0.03), order Bacteroidales id.913 (OR: 1.27, 95% CI 1.02–1.58, P=0.03), phylum Bacteroidetes id.905 (OR: 1.31, 95% CI 1.01–1.70, P=0.03), genus Senegalimassilia id.11160 (OR: 1.33, 95% CI 1.01–1.74, P=0.03)were associated with a higher risk of gingivitis. In contrast, family Porphyromonadaceae id.943 (OR: 0.67, 95% CI 0.47–0.95, P=0.02), genus Escherichia Shigella id.3504 (OR: 0.76, 95% CI 0.59–0.96, P=0.02), genus Ruminococcaceae UCG002 id.11360 (OR: 0.84, 95% CI 0.71–0.99, P=0.04), class Actinobacteria id.419 (OR: 0.82, 95% CI 0.67–0.99, P=0.04), was linked to a lower risk of gingivitis. All the MR analyses (WM and MR-Egger) present consistent results with the corresponding IVW analyses. In the weighted-median method, however, only the family Defluviitaleaceae id.1924 remained stable (OR: 1.29, 95% CI 1.00–1.65, P=0.04) (**Table 1**). In sensitivity analysis, the MR-Egger regression analysis showed no indications of directional pleiotropy (P-value for the intercept term > 0.05) (**Supplementary Materials Table S3**).

**Table 1:** MR estimates for the relationship between genetically instrumented gut microbiota and gingivitis.

Exposure	Method	OR	95%CI	p-value
class Actinobacteria id.419	IVW	0.82	0.67–0.99	0.04
	WM	0.81	0.61–1.07	0.14
	MR-Egger	0.69	0.39–1.21	0.22
class Bacteroidia id.912	IVW	1.27	1.01–1.58	0.03
	WM	1.63	1.03–2.56	0.05
	MR-Egger	1.28	0.95–1.74	0.10
family Defluviitaleaceae id.1924	IVW	1.30	1.08–1.57	0.005
	WM	1.29	1.00–1.65	0.04
	MR-Egger	1.23	0.65–2.33	0.53
family Porphyromonadaceae id.943	IVW	0.66	0.47–0.95	0.02
	WM	0.80	0.52–1.23	0.31
	MR-Egger	0.87	0.16–4.53	0.87
genus Defluviitaleaceae UCG011 id.11287	IVW	1.26	1.02–1.56	0.02
	WM	1.29	0.99–1.68	0.05
	MR-Egger	1.16	0.54–2.47	0.71
genus Escherichia Shigella id.3504	IVW	0.76	0.59–0.96	0.02
	WM	0.74	0.54–1.01	0.06
	MR-Egger	0.87	0.41–1.85	0.73
genus Ruminococcaceae UCG002 id.11360	IVW	0.84	0.71–0.99	0.04
	WM	0.82	0.65–1.03	0.09
	MR-Egger	0.74	0.48–1.15	0.20
genus Sellimonas id.14369	IVW	1.14	1.01–1.29	0.02
	WM	1.13	0.96–1.33	0.13
	MR-Egger	1.16	0.57–2.35	0.68
genus Senegalimassilia id.11160	IVW	1.33	1.01–1.74	0.03
	WM	1.38	0.99–1.92	0.05
	MR-Egger	1.66	0.58–4.71	0.40
order Bacteroidales id.913	IVW	1.27	1.01–1.58	0.03
	WM	1.63	1.03–2.56	0.05
	MR-Egger	1.28	0.95–1.74	0.10
phylum Bacteroidetes id.905	IVW	1.31	1.01–1.70	0.03
	WM	1.79	0.99–3.25	0.08
	MR-Egger	1.29	0.91–1.84	0.14

### 3.3 Sensitivity analyses and detection of pleiotropy

To minimize potential bias effects, pleiotropic analyses were conducted. No pleiotropies were detected in the IVs for the 11 mentioned taxa causally associated with gingivitis (P > 0.05). The conclusions gained further support through leave-one-out sensitivity analyses. Funnel plots indicated that causal associations were unlikely to be influenced by potential biases, given the symmetrical distribution of SNPs. Despite heterogeneous results, Cochran’s Q tests found no evidence of heterogeneity among the 11 taxa (P > 0.05, **Table 2**; **Supplementary Materials Table S6**). Additionally, MR-Egger intercept tests showed no indications of horizontal pleiotropy within these 11 taxa (**Supplementary Materials Table S4**). In summary, our MR analyses are confirmed to be reliable and robust. These results collectively suggest that the identified causal relationships between gut microbiota and gingivitis are likely mediated by the specified gut bacterial taxa.

**Table 2:** MR-Egger test for directional pleiotropy and heterogeneity.

Exposure	Intercept	p-value	Q	Q_p-value
class Actinobacteria id.419	0.01	0.54	12.9	0.45
class Bacteroidia id.912	-0.02	0.23	7.0	0.85
family Defluviitaleaceae id.1924	0.005	0.86	5.9	0.74
family Porphyromonadaceae id.943	-0.01	0.75	9.7	0.20
genus Defluviitaleaceae UCG011 id.11287	0.009	0.82	4.9	0.66
genus Escherichia Shigella id.3504	-0.01	0.71	2.3	0.96
genus Ruminococcaceae UCG002 id.11360	0.01	0.55	14.7	0.79
genus Sellimonas id.14369	-0.002	0.96	5.4	0.61
genus Senegalimassilia id.11160	-0.02	0.69	0.8	0.85
order Bacteroidales id.913	-0.02	0.23	7.1	0.85
phylum Bacteroidetes id.905	-0.02	0.28	3.9	0.91

### 3.4 Replication and meta−analysis

**Figure 4:**
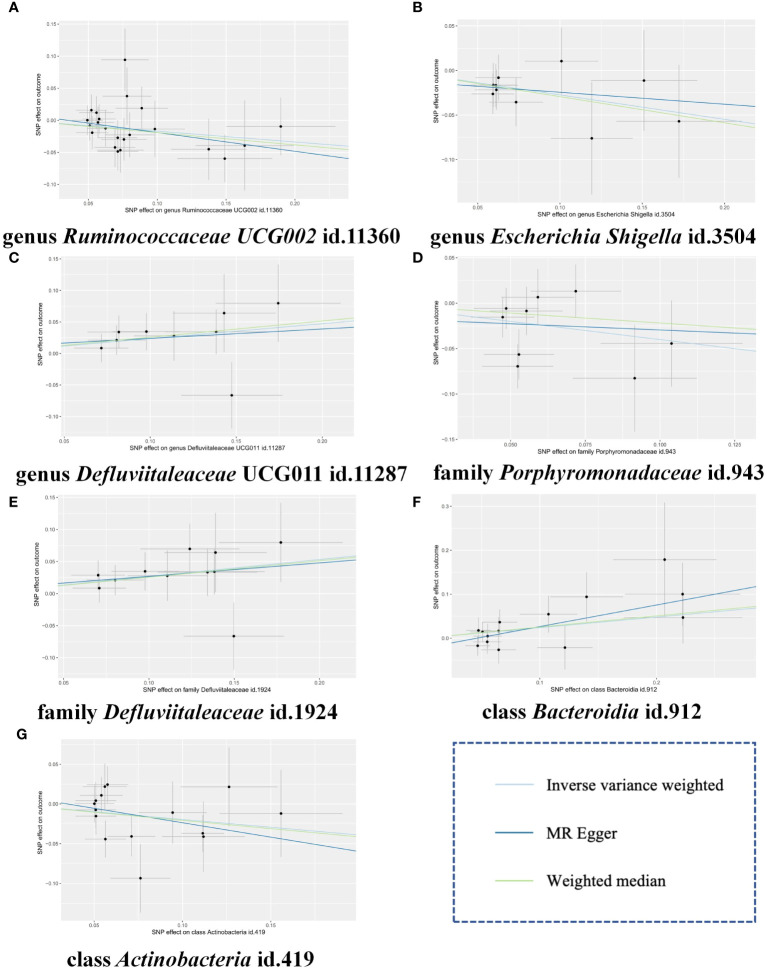
Scatter plots of the MR estimates for the significant causality of 7 gut microbiota taxa and the risk of gingivitis. **(A)** The causal effect of genus Ruminococcaceae UCG002 id.11360 on gingivitis; **(B)** The causal effect of genus Escherichia Shigella id.3504 on gingivitis; **(C)** The causal effect of genus Defluviitaleaceae UCG011 id.11287 on gingivitis; **(D)** The causal effect of family Porphyromonadaceae id.943 on gingivitis; **(E)** The causal effect of family Defluviitaleaceae id.1924 on gingivitis; **(F)** The causal effect of class Bacteroidia id.912 on gingivitis; **(G)** The causal effect of class Actinobacteria id.419 on gingivitis. The lines implying positive correlations moved diagonally upward from left to right, indicating a facilitative effect of gut microbiota on gingivitis. The horizontal and vertical lines indicated each correlation’s 95% confidence interval. The lines implying negative correlations move diagonally downward from left to right, indicating the inhibitory effect of gut microbiota on gingivitis. MR, Mendelian randomization; SNPs, single nucleotide polymorphisms.

**Figure 5:**
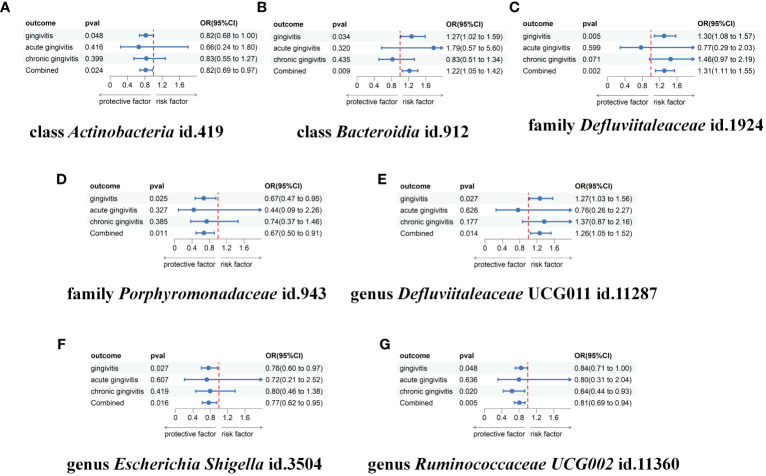
Meta-analysis of the causal associations between gut microbiota taxa and gingivitis, acute gingivitis and chronic gingivitis. **(A)** The meta-analysis of the causal associations between class Actinobacteria id.419 and gingivitis; **(B)** The meta-analysis of the causal associations between class Bacteroidia id.912 and gingivitis; **(C)** The meta-analysis of the causal associations between family Defluviitaleaceae id.1924 and gingivitis; **(D)** The meta-analysis of the causal associations between family Porphyromonadaceae id.943 and gingivitis; **(E)** The meta-analysis of the causal associations between genus Defluviitaleaceae UCG011 id.11287 and gingivitis; **(F)** The meta-analysis of the causal associations between genus Escherichia Shigella id.3504 and gingivitis; **(G)** The meta-analysis of the causal associations between genus Ruminococcaceae UCG002 id.11360 and gingivitis. OR, odds ratio; CI, confidence interval.

To further validate our findings, we conducted a replication analysis using the GWAS data for gingivitis (acute gingivitis and chronic gingivitis) from IEU OpenGWAS. As expected, similar trends were observed in certain metabolites when analyzing the gingivitis GWAS data from IEU OpenGWAS (**Supplementary Materials Table S7**). The combined analysis of the IEU OpenGWAS and FinnGen datasets revealed that a higher genetic susceptibility to class Actinobacteria id.419 (OR=1.22, 95% CI=1.05–1.41, P=0.009), family Defluviitaleaceae id.1924 (OR=1.31, 95% CI=1.11–1.55, P=0.002), genus Defluviitaleaceae UCG011 id.11287(OR=1.26, 95% CI=1.04–1.52, P=0.014), was associated with an increased risk of gingivitis. Conversely, a genetic predisposition to higher levels of class Actinobacteria id.419 (OR=0.82, 95% CI=0.69–0.97, P=0.024), family Porphyromonadaceae id.943(OR=0.67, 95% CI=0.49–0.91, P=0.011), genus Escherichia Shigella id.3504(OR=0.76, 95% CI=0.61–0.95, P=0.016), genus Ruminococcaceae UCG002 id.11360(OR=0.80, 95% CI=0.69–0.94, P=0.005) predicted a lower risk of gingivitis (**Figure 4**). In using the IEU OpenGWAS database, null estimates were observed in genus Senegalimassilia id.11160, order Bacteroidales id.913, phylum Bacteroidetes id.905, genus Sellimonas id.14369 with inconsistent directions (**Figure 5**).

### 3.5 Genetic correlation validation

Using LDSC, we found little evidence of genetic correlation between gingivitis and family Porphyromonadaceae id.943(r_g_ = -0.215, se = 0.271, P = 0.432), genus Defluviitaleaceae UCG011 id.11287(r_g_ = 0.015, se = 0.426, P = 0.256). Additionally, our findings indicate a suggestive genetic correlation between class Actinobacteria id.419(r_g_ = -0.302, se = 0.088, P = 0.002), class Bacteroidia id.912(r_g_ = 0.121, se = 0.103, P = 0.045), family Defluviitaleaceae id.1924(r_g_ = 0.352, se = 0.112, P = 0.036), genus Escherichia Shigella id.3504(r_g_ = -0.011, se = 0.182, P = 0.023) and gingivitis. Owing to limitations such as low heritability and sample size, genus Ruminococcaceae UCG002 id.11360 cannot be used for the above analysis (**Table 3**; **Supplementary Materials Table S8**).

**Table 3:** The genetic correlations between gut microbiota and gingivitis.

Trait1	Trait2	r_g_	SE	P-value
class Actinobacteria id.419	gingivitis	-0.302	0.088	0.002
class Bacteroidia id.912	gingivitis	0.121	0.103	0.045
family Defluviitaleaceae id.1924	gingivitis	0.352	0.112	0.036
family Porphyromonadaceae id.943	gingivitis	-0.215	0.271	0.432
genus Defluviitaleaceae UCG011 id.11287	gingivitis	0.015	0.426	0.256
genus Escherichia Shigella id.3504	gingivitis	-0.011	0.182	0.023

## 4 Discussion

In this investigation, a two-sample MR analysis was conducted to assess the potential causal association between gut microbiota and gingivitis. Our findings suggest an intricate interaction between gut microbiota and gingivitis. Specifically, three microbial were identified with an elevated risk of gingivitis, while four exhibited an association with a diminished risk. Our research findings bridge the knowledge gap regarding the potential role of gut microbiota in gingivitis and delineate specific taxonomic groups that may either facilitate or hinder the onset of gingivitis.

The correlation between gut microbiota and gingivitis has long been a subject of research interest (Byrd and Gulati, 2021). Traditional research methodologies face inherent challenges in fully elucidating the intricate relationship between gut microbiota and gingivitis. This complexity stems from the dynamic nature of both oral and gut microbiota, influenced by multifaceted factors such as diet, lifestyle, genetics, environment, medication, and disease. Moreover, the precise causal direction and underlying mechanisms governing the bidirectional influence of oral and gut microbiota, along with their impact on the inflammatory processes within the oral and intestinal mucosa, remain incompletely understood. Consequently, observational studies are susceptible to confounding variables and reverse causation, while experimental approaches may not capture the comprehensive temporal and spatial variations in microbiota and host response (Smith and Ebrahim, 2003). Investigating host genetic variation stands forth as a compelling and pivotal research domain (Chen Q. et al., 2023). To overcome these limitations, some researchers have proposed and applied novel methods to investigate the causal relationship between gut microbiota and gingivitis, such as Mendelian randomization, metabolomics, and machine learning. These methods can leverage the genetic and metabolic data of the microbiota and the host, and use statistical and computational techniques to infer the causal effects and interactions of the microbiota on the disease outcomes.

It is noteworthy that we discovered novel taxa with positive associations that have not been reported in prior literature. The study revealed a higher abundance of the genus Defluvitataeaceae on the tonsil surface in the hypertrophic group compared to the healthy group, indicating a potential association with tonsillar inflammation (Xu et al., 2021). Defluviataleaceae, like Defluvitataeaceae, also falls within the order Defluviatales; however, it is distinct from Defluvitataeaceae based on phylogenetic and phenotypic characteristics. It has been mentioned that some Defluviataleaceae bacteria can degrade aromatic compounds, such as phenol and benzoate, which are known to induce oxidative stress and inflammation in the brain (Talebi et al., 2021). Through our MR analysis, it can be concluded that reducing the number of family Defluviitaleaceae and genus Defluviitaleaceae UCG011 is beneficial for controlling gingivitis.

Utilizing whole-genome sequencing and bioinformatics analysis, the study identified that Bacteroidia, encompassing the order Bacteroidales, demonstrated notable diversity and abundance in the oral cavity (Caselli et al., 2020). Based on preceding investigations, it was noted that Bacteroidales represented one of the taxa exhibiting a marked adverse effect on gingivitis (Luo et al., 2023). However, conflicting outcomes emerge from an observational study, suggesting the anti-inflammatory properties of metabolites originating from the Bacteroidales group (Luo et al., 2023). Indeed, our MR study can overcome these challenges by providing validation from a genetic perspective, presenting clear evidence that the class Bacteroidia group contribute to the risk of gingivitis.

In addition to the previously mentioned three taxa promoting gingivitis, we also identified four gut microbiota taxa negatively associated with gingivitis, which are reported for the first time. Despite previous studies associating Porphyromonadaceae with inducing inflammation and tissue damage (Eisenstein, 2021), our investigation revealed that Porphyromonadaceae exhibits anti-inflammatory effects. The inconsistency among studies could be attributed to high individual variations in gut microbiota composition and the multifaceted nature of inflammatory diseases (Eckburg et al., 2005). Next, an article highlighted Escherichia Shigella and Ruminococcaceae UCG002 as generals that exhibited a significant increase in the intestinal microbiota of these patients with decompensated alcoholic cirrhosis. Additionally, it emphasized that short-chain fatty acids (SCFAs), produced by certain gut bacteria, may modulate the inflammatory response and liver function (Baltazar-Diaz et al., 2022). In conjunction with existing findings, our MR study suggests that preventive measures and control of gingivitis may be attainable by increasing the abundance of the genus Escherichia Shigella and genus Ruminococcaceae UCG002 through various interventions. A study underscored the significance of actinomycetes in the synthesis of bioactive compounds, including antibiotics, anticancer agents, anti-inflammatory agents, and enzymes (Selim et al., 2021), which may be the mechanisms of class Actinobacteria to reduce risks of gingivitis.

Considering the direct connection between the oral and gastrointestinal tracts and the extent to which disturbances in the oral microbiome affect the gut microbiome and subsequently gut stem cells, such as Lgr5, contributing to the systemic disease (e.g. liver cancer) development, remains unresolved (Barker and Clevers, 2007; Han et al., 2021; He et al., 2023), investigating the interaction between these microbiotas is of significant relevance (Uchiyama et al., 2019). Elucidating the specific contribution of distinct gut microbial taxa to gingivitis holds potential for enhancing prevention and control strategies. Despite efforts to understand the association between gingivitis and gut microbiota, no evidence supporting a causal effect has been proposed. Furthermore, the dysbiosis phenotype observed in the gut microbiota of gingivitis patients results from multifactorial influences, and the strain-specific changes in various microbiota taxa are inconsistent. Variability in the composition of the gut microbiota can be attributed to disparities in the staging of gingivitis, gender distribution, and ethnic composition across different study populations. These factors present challenges in establishing a specific causal relationship between gut microbiota taxa and the risk of gingivitis. Further, Oral disease treatment has been proven effective in alleviating inflammatory symptoms in patients with periodontitis and associated systemic diseases, notably including improvements in cirrhosis patients (Bajaj et al., 2018).This effectiveness prompts the investigation of whether such treatments could also impact other systemic disease (e.g. liver cancer) progression by modifying underlying biological pathways, potentially guiding the development of targeted periodontal interventions for the disease prevention. In short, the pivotal role of gut microbiota in gingivitis and overall health underscores the potential for investigating targets along the “oral-gut axis” to manage inflammatory disorders, which may involve immunological approaches to regulate intestinal microbiota (Zhou et al., 2023).

As stated earlier, MR is an optimal study design for assessing causal relationships between potential risk factors and diseases. Recently, multiple MR studies have probed nuanced risk factors associated with gingivitis. This study from Frontiers in Genetics used MR to examine the causal relationship between circulating cytokines and gingivitis. It found that interleukin 9 (IL9) had a positive causal relationship with gingivitis, and interleukin 17 (IL17) had a negative causal relationship with gingivitis (Huang et al., 2023). Through the examination of factors influencing gingivitis risk, MR studies inform the development of public health policies and clinical interventions to effectively reduce its incidence and societal burden. Certain factors previously implicated in gingivitis through epidemiological observational studies were found to lack a causal association with gingivitis according to MR studies, such as depression and psoriasis (Nolde et al., 2022; Yin et al., 2022). In contrast to earlier MR studies, our investigation is more comprehensive, delineating the causal impact of 418 gut microbiome taxa on gingivitis. Previous MR studies concentrated on fewer than 10 exposures of interest. We conducted a replication analysis to verify the robustness of the MR estimates using two independent datasets (IEU OpenGWAS and FinnGen), greatly enhancing the credibility of our results.

This study represents the forefront of MR investigations, utilizing extensive genetic data on gut microbiome and gingivitis to assess the potential causal relationship between gut microbiota and the risk of gingivitis. A notable strength of our study is the robust application of the MR method, effectively mitigating concerns related to reverse causal associations and confounding factors. Additionally, our MR study encompasses a broad population range at a relatively low cost, offering practical and compelling insights compared to conventional observational studies. Nonetheless, this study has some limitations that need to be acknowledged. Firstly, the majority of the GWAS participants were of European descent, which may limit the applicability of our results to other populations. Secondly, considering the biological plausibility and intricate pathobiology of gingivitis, along with the multifaceted nature of statistical processes, applying a stringent multiple-testing correction may be excessively conservative. Such an approach could potentially overlook strains that are partially causally correlated with gingivitis. Consequently, we decided not to implement multiple corrections (Zhang et al., 2022). Thirdly, our study targeted the elucidation of risk factors for gingivitis with the goal of facilitating comprehensive clinical intervention and reducing the incidence. Consequently, our focus was on examining the unidirectional influence of 418 gut microbiota taxa on gingivitis. Fourth, the precise mechanisms by which the aforementioned gut microbiota taxa influence the risk of gingivitis have not been comprehensively investigated in this study.

In conclusion, this study is the first to demonstrate a causal relationship between gut microbiota and gingivitis using MR analyses. It reveals the impact of specific gut microbiota taxa on the susceptibility to gingivitis, offering new perspectives for designing clinical interventions to prevent and treat gingivitis.

## Data availability statement

The original contributions presented in the study are included in the article/**Supplementary Material**. Further inquiries can be directed to the corresponding author.

## Author contributions

ZH: Conceptualization, Data curation, Formal Analysis, Investigation, Methodology, Project administration, Resources, Software, Validation, Visualization, Writing – original draft. CR: Writing – original draft. LS: Conceptualization, Funding acquisition, Methodology, Supervision, Validation, Writing – review & editing.
